# Improving homology‐directed repair by small molecule agents for genetic engineering in unconventional yeast?—Learning from the engineering of mammalian systems

**DOI:** 10.1111/1751-7915.14398

**Published:** 2024-02-20

**Authors:** Min Lu, Sonja Billerbeck

**Affiliations:** ^1^ Molecular Microbiology, Groningen Biomolecular Sciences and Biotechnology Institute University of Groningen Groningen The Netherlands

## Abstract

The ability to precisely edit genomes by deleting or adding genetic information enables the study of biological functions and the building of efficient cell factories. In many unconventional yeasts, such as those promising new hosts for cell factory design but also human pathogenic yeasts and food spoilers, this progress has been limited by the fact that most yeasts favour non‐homologous end joining (NHEJ) over homologous recombination (HR) as a DNA repair mechanism, impairing genetic access to these hosts. In mammalian cells, small molecules that either inhibit proteins involved in NHEJ, enhance protein function in HR, or arrest the cell cycle in HR‐dominant phases are regarded as promising agents for the simple and transient increase of HR‐mediated genome editing without the need for a priori host engineering. Only a few of these chemicals have been applied to the engineering of yeast, although the targeted proteins are mostly conserved, making chemical agents a yet‐underexplored area for enhancing yeast engineering. Here, we consolidate knowledge of the available small molecules that have been used to improve HR efficiency in mammalian cells and the few ones that have been used in yeast. We include available high‐throughput‐compatible NHEJ/HR quantification assays that could be used to screen for and isolate yeast‐specific inhibitors.

## Graphical abstract

Here we consolidate knowledge on chemical agents that affect DNA repair meechanisms and how they have been employed to enhance sucess in mammalian cells and some yeast.

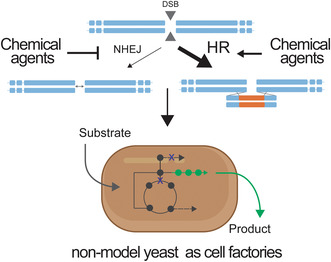

## INTRODUCTION

**FIGURE 1:**
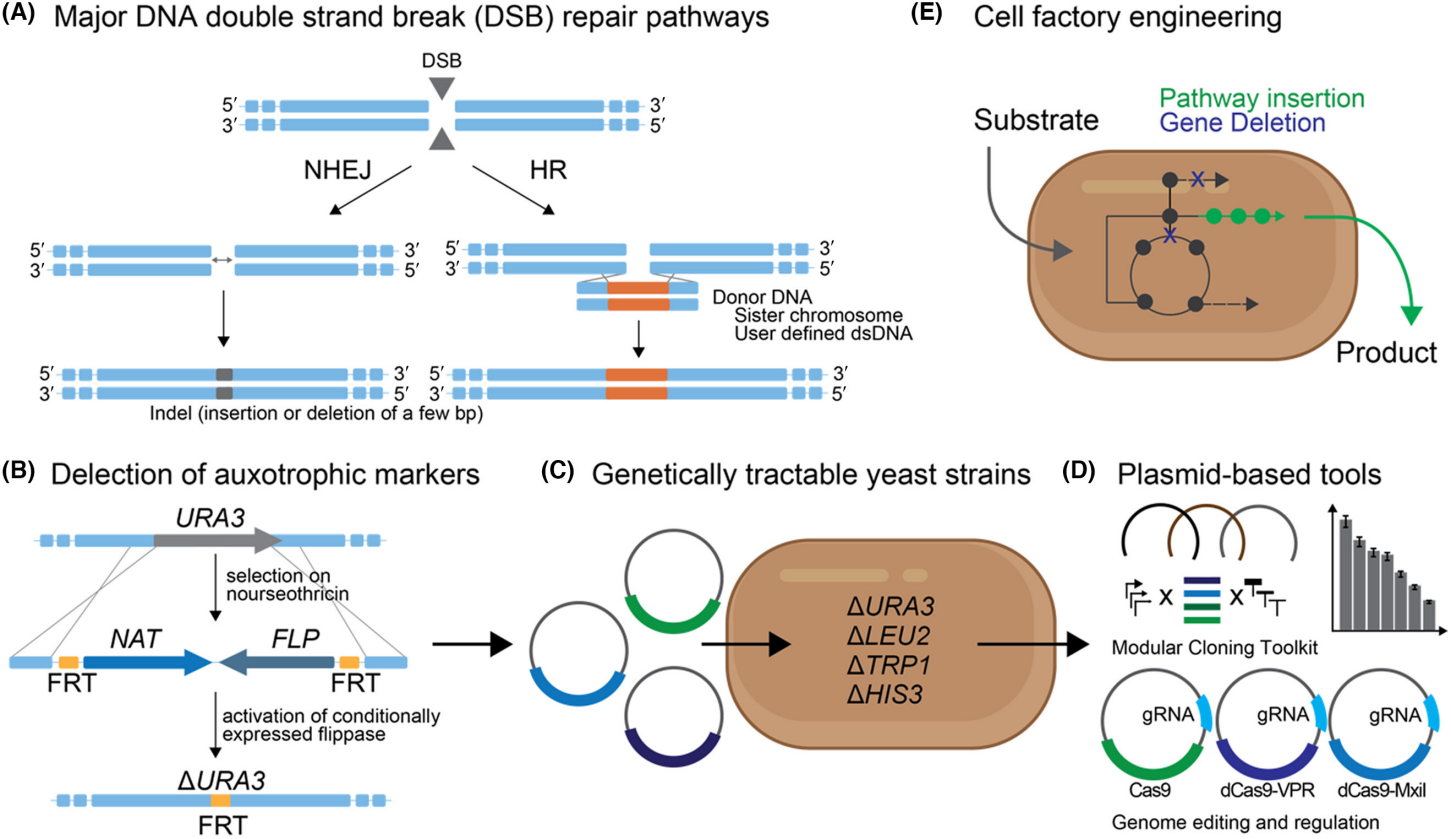
HR‐mediated repair of a DSB by a user‐designed donor DNA is a powerful tool to gain genetic access to unconventional yeast and turn them into cell factories. (A) Simple overview of the two major repair pathways that are activated after a DSB. A DSB can be induced anywhere in the genome by radiation or chemicals or it can be induced at a specific locus by genome editing tools such as CRISPR/Cas, TALENs or the I‐SceI endonuclease. Nonhomologous end‐joining (NHEJ) leads to error‐prone ligation of the broken DNA ends that can cause indels (insertions or deletions of a few base pairs) at the repaired site (see Dudášová et al., 2004) for a detailed overview in yeast). Homologous recombination (HR) uses a homologous donor DNA to repair the cut. This donor DNA can be derived from homologous sister chromatids or by creating a user‐defined fragment that encodes extensions (homologous arms) that are homologous to a given locus. (B) HR‐mediated repair can be harnessed to delete auxotrophic markers such as the URA3 gene (encoding for the enzyme Orotidine 5‐phosphate decarboxylase, which catalyses one step in the biosynthesis of pyrimidine ribonucleotides). The donor DNA usually encodes for an antibiotic selection marker NAT^R^ (giving resistance to nourseothricin) and a conditionally expressed flippase recombinase (FLP) that is used to eventually remove the NAT^R^ gene via recombination with reciprocal FRT sites and recycle it for the deletion of other auxotrophic markers (examples include (Moreno‐Beltran et al., 2021; Schwarzmuller et al., 2014). (C,D) One or multiple deleted auxotrophic markers allow for the transformation of a yeast with one or multiple plasmids that encode the deleted gene for selection on minimal media lacking the appropriate metabolite (Billerbeck et al., 2023; Brachmann et al., 1998). Auxotrophic markers are commonly used in yeast engineering as only a few antibiotic markers are available. Plasmids allow the building of modular cloning toolkits and the use of genome editing and genome regulation tools. (E) Modular gene expression, deletion, and insertion tools are essential for cell‐factory engineering via the insertion of expression‐balanced multi‐gene pathways and the deletion of interfering metabolic reactions.

DNA double‐strand breaks (DSB) induced by radiation, toxic chemicals or endogenous processes are the most serious DNA damage resulting in genome instability and cell death (Cannan & Pederson, 2016). To survive this injury, two major repair pathways are rapidly activated: non‐homologous ending joining (NHEJ) or homologous recombination (HR) (Figure 1A). NHEJ is active during the whole cell cycle and is regarded as an error‐prone pathway, causing the random insertion or deletion of bases and thus potentially disrupting open reading frames. HR is a more precise repair mechanism where broken ends are ligated with the help of homologous donor DNA. HR works more efficiently in the S and G2 phases of the cell cycle, where homologous sister chromatids are available (Saha et al., 2017). Even though there is competition between NHEJ and HR repair (Azhagiri et al., 2021), NHEJ always acts kinetically faster and dominates in mammalian cells and most yeast species.

Two exceptions are the yeasts *Saccharomyces cerevisiae* and *Schizosaccharomyces pombe* which, at least the well‐established laboratory strains, favour HR during all stages of the cell cycle. The fact that *S. cerevisiae* primarily employs HR for DSB repair has turned this yeast into a living machine for DNA assembly up to genome size (Gibson et al., 2008) and has facilitated the development of simple, markerless and multiplexable genome editing tools that enabled the refactoring of entire pathways and the implementation of synthetic metabolism (Malci et al., 2020).

Although *S. cerevisiae* has been extensively engineered for commercial manufacturing (Kavscek et al., 2015; Nielsen, 2019), various of the other 1500 known yeast species have gained attention as potential cell factories. This is because these unconventional yeasts show a natural capacity to produce desired commodity chemicals, a broad capacity to use various substrates and their often more robust process characteristics. Recent reviews (Cai et al., 2019; Park et al., 2022) highlight examples such as the oleaginous yeast *Yarrowia lipolytica* for lipid production, *Kluyveromyces marxianus* for its thermotolerance and broad substrate utilisation, *Scheffersomyces stipitis* for its natural capacity for xylose utilisation and production of aromatic compounds, and the methylotrophic yeasts *Hansenula polymorpha* and *Pichia pastoris* for their extremely efficient heterologous protein secretion and glycosylation ability.

Further, besides the field of biotechnology, several unconventional yeasts are pathogens to humans or food spoilers. Examples include *Candida albicans*, *C. tropicalis, C. auris*, *C. glabrata*, *Zygosaccchoromyces rouxii* and *balii or Brettanomyces bruxiliensis*.

Infections caused by opportunistic pathogenic yeast can be detrimental especially to immunocompromised individuals, resulting in 80,000–2.3 million cases every year (Gabaldon et al., 2019). In addition, antifungal resistance has been rising and multi‐resistant strains of *C. glabrata* and *C*. *auris* have emerged (Fisher et al., 2016; Gabaldon et al., 2019).

The genetic tractability of unconventional yeast is thus not only fundamental for metabolic engineering and gaining access to new cell factories for a sustainable bio‐economy but also for answering basic biological questions about pathogenicity and host‐microbe interactions for promoting understanding of antifungal mechanisms of action and ultimately developing novel antifungal drugs.

Homology‐directed repair is an essential tool that allows access to the genetics of a given host at all stages, from the use of plasmids to genome editing (Figure 1B,C) and its absence in a cell can make simple engineering difficult. For example, HR is a powerful (almost essential) tool for making strains accessible for plasmid transformation by deleting commonly used auxotrophic markers. The use of plasmids is then the first step in developing well‐characterised modular cloning toolkits (Billerbeck et al., 2023; Lee et al., 2015), which have proven useful for cell factory engineering (Besada‐Lombana et al., 2018) and for using plasmid‐encoded genome editing or regulatory tools, such as CRISPR/Cas9 and CRISPRi/a. Beyond plasmids, HR‐mediated DSB repair via donor DNA is an essential requirement for precise genome editing such as the insertion of pathways or deletion of enzymatic functions (Figure 1D): fundamental steps in cell‐factory engineering. Of note, multiplexing of genome editing in *S. cerevisiae* has been explicitly easy because relatively short homology arms (~40 bps) are sufficient to mediate HR, and those can be encoded on primers, allowing a user to cheaply generate any donor DNA by one‐step PCR. In several other yeasts, HR can only be detected when longer homology arms are provided (~500–1000 bps) (Ji et al., 2020; Moreno‐Beltran et al., 2021; Schwarzmuller et al., 2014), which requires more laborious fusion or extension PCRs.

Due to the essential nature of HR for genome engineering and the compromising fact that HR is outcompeted by NHEJ in most non‐*S. cerevisiae* yeasts and also in mammalian cells (Cai et al., 2019; Raschmanova et al., 2018; Wagner & Alper, 2016), several efforts have focused on enhancing repair bias towards HR by either impairing the NHEJ machinery, enhancing the HR machinery or harnessing the cell‐cycle S/G2 phase‐specific HR dominance. Most strategies are genetic: many studies in yeast and mammalian cells have demonstrated that deletion of the Ku70/80 complex, an important and conserved component of the NHEJ machinery, leads to a much higher HR‐mediated gene knock‐in (KI) efficiency (Arras & Fraser, 2016; Kooistra et al., 2004; Nambiar et al., 2019; Nayak et al., 2006; Ninomiya et al., 2004). Further, overexpression of the *S. cerevisiae* ScRad51 or ScRad52—two key components of the very efficient HR machinery in *S. cerevisiae*—enhanced repair via HR in mammalian cells (Shao et al., 2017; Vispe et al., 1998) and yeast (*Y. lipolytica*) (Ji et al., 2020). Other studies combined the overexpression of HR‐related proteins and down‐regulation or deletion of NHEJ‐related proteins (an approach called recombination machinery engineering) to enhance precise genome engineering in the yeasts *Pichia pastoris* and *Ogataea* (*Hansenula*) *polymorpha* (Cai et al., 2021; Gao et al., 2021). In addition, a Geminin‐based tag has been developed that restricts Cas9 expression and thus DSB induction to the S/G2 cell cycle phase and this tag has been successfully employed in human and yeast cells with enhanced HR efficiency (Ploessl et al., 2022). Although these genetic methods improve the low HR efficiency, they require a priori access to the host's genetics, which is often not available. This is because plasmid selection in yeast relies on auxotrophic markers due to the scarcity of well‐working antibiotic selection markers and the physiological effects of antibiotics on cell function (Pronk, 2002) that complement genomically disrupted biosynthetic pathways for certain amino acids (such as Uracil or Tryptophan), which require a priori engineering of the yeast (Figure 1C). Also, the deletion of the Ku70/80 affects cell growth and even telomere instability (Ploessl et al., 2022; Sui et al., 2020).

An alternative is the use of small molecules that transiently bias repair to HR. The effects of these small molecules mirror the effects of the genetic methods: they directly inhibit NHEJ‐related proteins, enhance the activity of HR‐mediating proteins or synchronise and arrest the cell cycle in the S/G2 phase. Small molecules can be purchased at an affordable cost (Supplementary Table S1), and employed without the need for laborious plasmid constructions or genetic changes, further, their action is transient and reversible such that wild‐type genotypes for functional studies or cell factory engineering can be maintained.

Small molecules that inhibit NHEJ have been widely developed for cancer therapy (Hengel et al., 2017) and the same molecules have subsequently been successfully employed in mammalian cell engineering (Chen et al., 2022; Shams et al., 2022). Given the fact that most proteins involved in NHEJ and HR are conserved (Dudášová et al., 2004; Li & Heyer, 2008), it seems worthwhile exploring if these small molecules can be successfully applied in yeast engineering, and/or how chemical agents optimised for yeast can be identified. Mammalian studies already indicate that these molecules can cross the cytosolic membrane and gain access to the nucleus. Given their small molecular weight (Supplementary Figure S1), they likely also diffuse through the yeast cell wall which is reported to be quite permeable to large molecules including proteins in actively growing cells (Denobel & Barnett, 1991).

In this review, we introduce small molecules that have been successfully applied in mammalian cells and feature the few studies that have tested these molecules in yeast. We summarise HR/NHEJ quantification assays that could be repurposed to find new or modified molecules that are specifically yeast‐specific molecules.

As a note, the reported efficiencies of the various chemicals in human cells are difficult to compare given their dependence on cell line, used concentration, varying length of homology arms and loci (Shams et al., 2022), and the fact that no standardised assays for measuring ‘precise engineering’ exist, but assays have rather been geared towards answering a specific question or meeting a specific goal of a given study.

## INHIBITORS OF NHEJ

**FIGURE 2:**
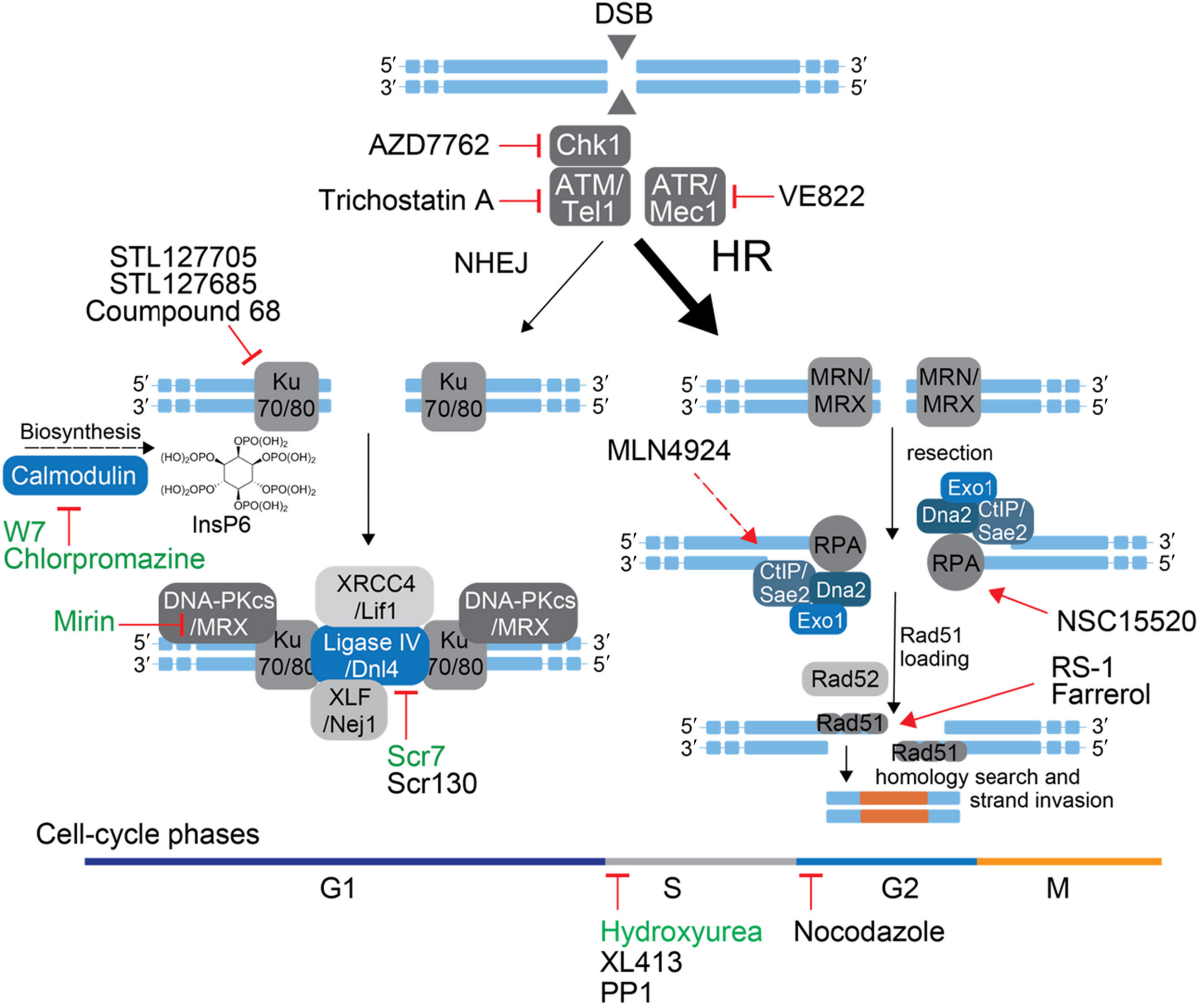
Small molecules described or anticipated to target key proteins of NHEJ and HR in humans and in yeast as well as to interfere with cell‐cycle progression. The figure gives a simplified overview of the proteins involved in each pathway. For readability, some proteins and protein interactions are not shown. Small molecule inhibitors or activators that have been tested in yeast are shown in green. An overview of the molecules and their targets can also be found in Table 1. The molecule MLN4924 indirectly activates CtIP—indicated by a dashed arrow—by inhibiting the NEDD8‐activating enzyme (NAE) and thus inhibiting the neddylation of CtIP.

In eukaryotes, repair of a DSB by NHEJ starts with the Ku70/Ku80 heterodimer—the DNA‐dependent protein kinase regulatory factor—binding the two loose DNA ends, acting as a structural linker that protects them from degradation (Dudášová et al., 2004; Figure 2). In humans, the Ku70/Ku80 heterodimer then recruits the catalytic subunit DNA‐PKcs, which is thought to induce conformational changes that allow end‐processing enzymes to access the DNA ends. *S*. *cerevisiae* does not encode a homologue of DNA‐PKcs. Instead, a complex consisting of Mre11, Rad50 and Xrs2 (short MRX) is thought to perform this function; a similar complex also participates in mammalian NHEJ (Mre11/Rad50/NBS1; short MRN). In both species, the process of NHEJ is completed by the DNA ligase IV‐XRCC4 complex, which ligates the broken ends of the DNA, depending on the overhangs of the cut, this can be error prone or clean (Dudášová et al., 2004). Most small‐molecule inhibitors developed for mammalian cells target one of the four major NHEJ components: the Ku70/80 heterodimer (directly or indirectly), the DNA‐PK catalytic subunit, Mre11 (parts of the MRN/MRX complex) or Ligase IV, three of which are shared amongst yeast and mammalian NHEJ. Here we summarise those molecules that have been used in mammalian engineering (Chen et al., 2022; Shams et al., 2022)—some successful, some showing no effect, some with varying results in different cell lines—and yeast (Table 1, Supplementary Table S2 and Figure 2).

**TABLE 1:** Small molecules described or anticipated to improve HR‐mediated genome editing in mammalian cells and which have potential targets in yeast.

Small molecules	Target	Target in yeast	Yeast species used for testing	Effect in HR in yeast	Reference
*Inhibition of NHEJ*					
STL127705	Ku70/80	yKu70/80	–	–	Guo et al. (2022), Weterings et al. (2016)
STL127685			–	–	Riesenberg and Maricic (2018)
Compound 68			–	–	Gavande et al. (2020), Moreno‐Beltran et al. (2021)
W7	Calmodulin/Ku‐cofactor InsP6	Calmodulin/InsP6	*Cryptococcus neoformans*; *Metschnikowia pulcherrima*	4‐fold increase; 2% increase	Arras and Fraser (2016), Moreno‐Beltran et al. (2021)
Chlorpromazine			*C. neoformans*	3‐ fold increase	
Scr7	Ligase IV	Dnl4	*C. neoformans*	–	Anuchina et al. (2023), Arras and Fraser (2016), Gerlach et al. (2018) Hu et al. (2018), Maruyama et al. (2015), Song et al. (2016), Zhang et al. (2018)
Scr130			–	–	Ray, Raul et al. (2020)
Mirin	Mre11	Mre11	*C. neoformans*	2‐fold increase	Arras and Fraser (2016)
VE‐822	ATR	Mec1	–	–	Ma et al. (2018)
AZD7762	Chk1	ScChk1	–	–	Ma et al. (2018)
Trichostatin A	ATM	Tel1	–	–	Riesenberg and Maricic (2018)
MLN4924	NAE	Neddylation	–	–	Riesenberg and Maricic (2018)
*Enhancement of homologues recombination*					
RS‐1	Rad51	Rad51	–	–	Lamas‐Toranzo et al. (2020), Pinder et al. (2015) Song et al. (2016)
Farrerol	Rad51	Rad51	–	–	Zhang et al. (2020)
NSC15520	RPA70	RPA 70	–	–	Riesenberg and Maricic (2018)
*Cell‐cycle synchronisation*					
Hydroxyurea	S phase	S phase	*Candida intermedia*	70% efficiency with split‐marker approach	Peri et al. (2023)
			*Arxula adeninivorans* *S. cerevisiae, Kluyveromyces lactis, Pichia pastori*	1.2‐ to 8‐ fold increase	Tsakraklides et al. (2015)
			*Y*. *lipolytica*	100% HR efficiency with disruption of Ku70	Jong et al. (2018)
			*M. pulcherrima*	4% increase	Moreno‐Beltran et al. (2021)
XL413			–	–	Shy et al. (2022), Wienert et al. (2020)
PP1			*S. cerevisiae SK1*		Wan et al. (2006)
Nocodazole	G2 phase	G2 phase	–	–	Eghbalsaied and Kues (2023), Katada et al. (2012), Lin et al. (2014)

*Note*: For simplicity, the *S. cerevisiae* gene name was chosen when indicating the yeast target. Based on the *S. cerevisiae* target, homologues in non‐conventional yeast can be identified.

### Inhibitors of the Ku70/80 heterodimer showed compound and cell‐line dependent enhancement of HR‐mediated knock‐in (KI) rates in human cells but they have not been tested in yeast

Mammalian and yeast Ku70/80 are conserved and its deletion has shown significant enhancement of HR‐mediated genome editing in many species, including yeast (Arras & Fraser, 2016; Kooistra et al., 2004; Nayak et al., 2006; Ninomiya et al., 2004). A computational small‐molecule screen against a potential binding pocket within the human Ku70/80 heterodimer yielded the small molecule STL127705, which showed strong inhibition of Ku70/80 binding to DNA and inhibition of the Ku‐dependent activation of the DNA‐PKcs kinase in vitro and in human cell lines, as well as higher cellular susceptible to radiation, indicating a clear potential to diminish DSB repair by NHEJ (Guo et al., 2022; Weterings et al., 2016). STL127705 has not been tested in genome editing studies. However, its derivative STL127685 was tested for CRISPR‐based genome editing and exhibited no significant effect on targeted nucleotide substitution (TNS) efficiency in hiPSC lines (Riesenberg & Maricic, 2018), but has not been tested in yeast. An independent computational structure‐guided small‐molecule screen targeting the DNA‐binding activity of the Ku70/Ku80 heterodimer (so‐called Ku DNA binding inhibitors, short Ku‐DBi's) yielded compounds 68, 149, and 322 which inhibited the Ku‐DNA interaction in vitro (Gavande et al., 2020). A further study showed that multiple Ku‐DBi's sensitised non‐small cell lung cancer (NSCLC) cells to DSB‐inducing agents (Mendoza‐Munoz et al., 2023). The utility of various Ku‐DBi's was also tested via CRISPR‐based genome editing increasing the KI efficiency in mouse and human cells (Gavande et al., 2020). Neither of the Ku‐DBi's has been tested in yeast. Given most Ku70/80 inhibitors have been developed and optimised by computational design, and given structural information of the human and *S. cerevisiae* Ku70/80 heterodimer (PDBs 1JEQ, 1JEY, 5Y58) are available, docking studies using AlphaFold predicted Ku70/80 structures from various non‐conventional yeast could potentially yield insights into their binding affinity or guide the design of better fitting inhibitors.

### InsP6‐depletion via calmodulin inhibition enhanced HR‐mediated KI efficiencies in the two tested yeast species in a species‐dependent manner

Inositol hexakisphosphate (InsP6) is a potent cofactor of the Ku70/Ku80 activity in NHEJ. InsP6 binds to Ku70/Ku80 and induces a conformational change required for Ku70/80 mobility and migration into the nucleus where it can participate in DNA end‐joining activity (Byrum et al., 2004). The biosynthesis of the small molecule InsP6 requires calmodulin. The compound W7—short for N‐(6‐aminohexyl)‐5‐chloro‐1‐naphthalenesulfonamideis—and Chlorpromazine are both calmodulin inhibitors that were shown to deplete InsP6 in Hela cells and to inhibit Ku mobility (Byrum et al., 2004). Thus W7 and Chlorpromazine were considered potent agents for augmenting HR efficiency (Byrum et al., 2004), but to the best of our knowledge, these compounds have not been tested in human genome editing. The high level of conservation between human and yeast calmodulin (Davis & Thorner, 1989), inspired the testing of their effect on genome engineering in two yeast species: in *Cryptococcus neoformans* W7 increased HR‐based KI rates by 3‐ to 5‐fold and chlorpromazine showed a 2.5 to 4‐fold enhancement both depending on the locus and the concentration (Arras & Fraser, 2016). Both molecules subsequently allowed the authors to achieve routine homology‐directed gene deletions with >50% success rate across other loci (Arras & Fraser, 2016). In one strain of *Metschnikowia pulcherrima*, W7 showed a very moderate 2% increase in HR‐mediated integration rate in a concentration‐dependent manner, chlorpromazine was not tested (Moreno‐Beltran et al., 2021).

### Ligase IV inhibition showed variable effects in human cell lines and has been tested in one yeast species with no effect

Ligase IV connects broken DNA ends in the final step of NHEJ. A computational docking screen using a homology model of Ligase IV yielded the potential inhibitor molecules Scr7 and several derivates that subsequently showed to block NHEJ in vitro and to decrease cancer progression in vivo (Srivastava et al., 2012). Although controversial results exist about its activity and specificity (Greco et al., 2016), Scr7 was successfully tested to increase CRISPR‐based KI rates up to 19‐fold in various human/mammalian cell lines and mouse zygotes (Maruyama et al., 2015). Independent studies showed 1.7 and 3‐fold increase in KI rates in different cancer cell lines (Anuchina et al., 2023; Hu et al., 2018) and up to 15% increase in zebrafish embryos (Zhang et al., 2018) while other studies report no effect on gene insertion efficiencies (Gerlach et al., 2018; Song et al., 2016). Notably, all studies use different cell lines, loci and protocols (Ray, Vartak, et al., 2020). The efficacy of Scr7 was also determined in the pathogenic yeast *C. neoformans* (in the same assay as W7 and Chlorpromazine), but no significant increase in KI rate was observed (Arras & Fraser, 2016). A derivative of Scr7, Scr130, with increased ligase IV specificity, and a 20‐fold increased inhibitory coefficient was developed, that effectively inhibited NHEJ in cancer cells (Ray, Raul, et al., 2020) but that has not been tested yet in genome editing studies.

### Inhibition of Mre11 with Mirin reduces NHEJ in mammalian cells and enhanced HR in the one tested yeast species

Mre11 is part of the Mre11/Rad50/NBS1 (MRN) complex in humans and the Mre11/Rad50/Xrs2 (MRX) complex in yeast cells. Human and yeast Mre11 are homologues. The MRN/MRX complex is multifunctional and possesses 3′ to 5′ dsDNA and ssDNA endonuclease, ssDNA exonuclease and hairpin cleavage activities, which have essential roles in both repair pathways, NHEJ and HR (Figure 2). A chemical docking screen using the Mre11 3D structure from the eubacterium *Thermotoga maritima* as a guide identified the molecule Mirin as an inhibitor of MRN/MRX (Dupre et al., 2008). Studies with Mirin analogs, designed to specifically inhibit either its exonuclease or endonuclease activity, show that Mre11 plays a critical role in the cellular choice over a repair pathway (Shibata et al., 2014). A CRISPR‐assisted DSB‐repair screen showed that Mirin reduced NHEJ and its alternative pathways at various cut sites (Taheri‐Ghahfarokhi et al., 2018), but to the best of our knowledge, it has not been tested in genome homology‐directed engineering studies in human cells. However, it was tested in the yeast *C. neoformans* and showed a >30% increase in KI rates (Arras & Fraser, 2016).

While Mre11‐inhibition by Mirin seems to enhance HR in yeast, it was also reported that a Cas9‐Mre11 fusion protein (thus overexpression) improved HR in human cells (Tran et al., 2020). As such, Mre11 inhibition and its overexpression show reciprocal results, and more detailed knowledge about Mre11 function (especially in in yeast) would be required to clarify these results. Still, given the promising results of Mirin in yeast, we believe it is worthwhile including this compound in chemical screens.

### Several inhibitors that target proteins involved in cellular decision‐making over NHEJ versus HR have been successfully employed in human cells but not tested in yeast

The protein targets ATR, ATM and Chk1 are important participators in the DNA damage response (Jin & Oha, 2019; Lei et al., 2022; Wang & Zhao, 2021). Further, 53BP1 plays a key role in the choice of repair mechanism, specifically facilitating NHEJ over HR by blocking end resection and inhibiting the recruitment of BRCA1 (Canny et al., 2018). In G1 phase, ATM (yeast homologue: Tel1) and ATR (yeast homologue: Mecl) mediate the phosphorylation of Chk1 (yeast homologues: Chk1), which facilitates the accumulation of 53BP1 at DSB ends, therefore NHEJ is initiated. However, in S and G2 phase, the recruitment of 53BP1 is inhibited by the phosphorylation of Chk1, instead BRCA1 accumulates at broken ends, leading to HR (Lei et al., 2022). In a small‐molecule screen, Ma et al. identify the molecules VE‐822 (inhibitor of ATR) and AZD7762 (inhibitor of Chk1) that increased CRISPR‐Cpf1‐mediated KI rates by 5.9‐ and 2.7‐fold, respectively, in human pluripotent stem cells (Ma et al., 2018). Trichostatin A (inhibitor of ATM) increased the targeted nucleotide substitution efficiency by 1.5‐ to 2.2‐fold in hiPSC lines when using a Cas9n approach (Cas9n is a nickase that introduces a single strand break, when used with two reciprocal gRNA it in their sum nicks both strands) but not when using regular Cas9 for DSB induction (Riesenberg & Maricic, 2018).

Another control point in decision‐making over repair pathways is protein neddylation. In neddylation, the small peptide NEDD8 gets conjugated to a protein to target it for proteasomal degradation (Maghames et al., 2018). Within DSB repair regulation, it was shown that neddylation inhibits CtIP‐mediated resection and biases repair towards NHEJ while inhibition of neddylation changes the normal repair profile towards an increase in HR (Ceppi et al., 2020; Jimeno et al., 2015). NEDD8‐activating enzyme (NAE) is an essential regulatory component of the NEDD8 conjugation pathway and the small molecule MLN4924 was identified as a potent and selective inhibitor of NAE (Soucy et al., 2009). MLN4942 very minimally enhanced precise genome editing by 1.1 to 1.3‐fold, but it was shown to work additive in combination with other small molecules when mixed (Riesenberg & Maricic, 2018) (see Point 5).

## HR ACTIVATORS

A detailed overview of homologous repair in yeast and humans can be found here (Li & Heyer, 2008). In short, after the detection of a double‐strand break (DSB), the MRN/MRX complex is loaded onto the DNA ends, and subsequently several helicases and nucleases, including Mre11, Sae2, Exo1, Dna2 and Sgs1 resect the DNA ends to create 3′ overhangs (Figure 2). RPA‐coated single‐stranded DNA overhangs bind Rad52 and drive Rad51 filament formation (Ma et al., 2017). Rad51 catalyses the key reactions that typify HR: homology search and DNA strand invasion. As Rad51 is a major player in HR, most current chemical strategies in mammalian cells target this protein. To the best of our knowledge, none of the HR enhancers has been tested in yeast.

### Stimulation of Rad51 enhances HR in mammalian cells but has not been tested in yeast

Overexpression of the *S. cerevisiae* Rad51 gene was shown to enhance HDR in mammalian cells (Vispe et al., 1998), and several small molecules that enhance its function have been reported.

RS‐1 was discovered in an HTP chemical library screen to enhance human Rad51 ssDNA binding activity in a wide range of biochemical conditions (Jayathilaka et al., 2008) and RS‐1 subsequently showed to increase HR‐mediated KI rates in various studies: 3 to 6‐fold in HEK293A cells dependent on locus and transfection method (Pinder et al., 2015), 5‐fold in rabbit embryos (Song et al., 2016) and also a significant increase in bovine embryos was observed (Lamas‐Toranzo et al., 2020).

Another Rad51 enhancer is the molecule Farrerol, which was isolated in a screen assaying CRISPR‐mediated HR rates exposed to a small molecule library from herbs used in traditional Chinese medicine. The authors show that Farrerol likely acts via accelerating the recruitment of Rad51 to broken DNA ends and enhanced the insertion rate 2 to 2.8‐fold in various human and mouse cell lines, performing better than Scr7 and RS‐1 which were tested in parallel (Zhang et al., 2020).

### A small molecule that potentially enhances the availability of RPA minimally enhances HR in mammalian cells but has not been tested in yeast

The replication protein A (RPA) is an essential player during HR by binding to single‐stranded DNA and initiating DNA end resection, it accumulates during S and G2/M phases (Kibe et al., 2007; Yamane et al., 2013). However, p53 can sequester RPA and its binding to ssDNA, there is an evidence that p53 when bound to RPA suppresses HR (Romanova et al., 2004). But the detailed mechanism remains to be explored. During an HTP chemical screen for inhibitors of the replication protein A (RPA), the small molecule NSC15520 was identified (Glanzer et al., 2011). NSC15520 prevents the association of RPA with p53, but does not impedes the binding of RPA to ssDNA. RPA provides the protection for ssDNA and promotes the recruitment of Rad51 with the help of Rad52 to ssDNA, therefore HR outcompetes NHEJ (Dueva & Iliakis, 2020; Glanzer et al., 2011). In analogy to the small molecule MLN4942 (neddylation inhibitor), NSC15520 enhanced precise genome editing only minimally by 1.3 to 1.4‐fold, but it worked additively in combination with other small molecules (Riesenberg & Maricic, 2018) (Point 5).

## CELL‐CYCLE SYNCHRONISERS

The fact that HR predominates in the S and the G2 phases of the cell cycle builds an opportunity to obtain a higher HR efficiency by reversibly arresting cells in one of the two phases.

### S‐phase arrest via Hydroxyurea (HU) increases the efficiency of HDR‐mediated genome editing in various yeast species but not in the one tested human cell line

HU leads to S‐phase arrest by inhibiting the enzyme ribonucleoside diphosphate reductase, thereby depleting cells of deoxyribonucleotides and thus limiting de novo DNA synthesis, leading to stalled replication forks and S‐phase arrest (Madaan et al., 2012). HU has successfully been used to increase homology‐directed gene integration in yeast: The HR efficiency was increased in a species‐dependent manner by 1.2‐ to 8‐fold in *Arxula adeninivorans, S. cerevisiae, Kluyveromyces lactis*, and *Pichia pastoris* even when using short homology arms (~50 bp). In *Candida intermedia* integration rates were boosted from 1% to 70% using a split‐marker genome editing approach (Peri et al., 2023). In *Y. lipolytica* KI rates at 15 loci showed a large range of enhancements, from 4 to 96% for different loci (Tsakraklides et al., 2015). In a separate study in *Y. lipolytica*, HU increased integration rates to up to 100% using relatively short homology arms of 100 bps, when Ku70 was additionally deleted (Jong et al., 2018). However, only a 4% increase in HR frequency was observed in *M. pulcherrima* (Moreno‐Beltran et al., 2021). HU showed no improvement in HR‐mediated genome engineering in HEK293T cells (Lin et al., 2014).

### S‐phase arrest via CDC7 inhibitors enhances genome integration in various mammalian cells but has not been tested in yeast

Cell division cycle 7‐related protein (Cdc7) kinase is a key regulator in the initiation of DNA replication and important for cell cycle progression into the G1 phase, inhibition of CDC7 leads to S‐phase arrest (Masai & Arai, 2002). Wienert et al. performed a screen using commercially available small molecule inhibitors of important cell‐cycle proteins, including CDC7, and found that XL413, a known CDC7 inhibitor, increased CRISPR‐mediated HR up to 3.5‐fold across multiple loci and in multiple cell lines, such as primary T cells, Hela, and HSPCs (Wienert et al., 2020). They compared efficiency with other inhibitors, and in their work, XL413 performed better than other molecules discussed herein, such as Scr7 or RS1 (Wienert et al., 2020). In an independent study, XL413 increased HR‐mediated gene insertion efficiency by 46% in primary human T cells (Shy et al., 2022). Despite CDC7 being strongly conserved across eukaryotes (Masai & Arai, 2002), XL413 has not been tested in yeast.

Further of note, the molecule PP1 was demonstrated to reversibly inhibit CDC7 activity in yeast and arrest cells in the S phase but has not been tested in genome engineering studies (Wan et al., 2006).

### Other cell cycle synchronisers enhance efficiencies in human cells but have not been tested in yeast

Lin et al. tested six known reversible chemical inhibitors synchronising HEK293T cells at the G1, S and M phases. They found aphidicolin (early S arrest) and nocodazole (G2 arrest) to enhance integration rates from 9% to almost 30% (Lin et al., 2014). Nocodazole had also been found to enhance HR‐mediated gene integration 2‐fold in HEK293T cells in an earlier non‐CRISPR‐based genome engineering study (Katada et al., 2012), and later Eghbalsaied et al. showed that Nocodazole increased HR‐mediated integration rate by 21% in a murine embryonic fibroblast cell line (Eghbalsaied & Kues, 2023). Nocodazole binds to beta‐tubulin and disrupts microtubule assembly/disassembly dynamics, impairing the formation of the metaphase spindles during the cell division cycle. This prevents mitosis by inducing a G2/M‐phase arrest (Jordan et al., 1992). Nocodazole has not been explored in yeast.

## COMBINATIONS OF CHEMICAL INHIBITORS AND ACTIVATORS

In addition, the combination of several small molecules with different modes of action has been successfully explored in mammalian cells. Riesenberg et al. showed that the so‐called CRISPY mix (a combination of the molecules NU7026, Trichostatin A, MLN4924, and NSC15520) had additive effects on precise editing efficiencies in different human cell lines (iPSCs and hESCs) and across three loci when using a double nicking strategy with Cas9n. In combination, the mix reached up to 50% precise nucleotide exchanges when compared to 10%–30% for single molecules. The CRISPY mix thereby combines NHEJ inhibitors and HR activators (Figure 2): It inhibits DNA‐PKcs with NU7026 (no homologue in yeast), enhances PKA with NSC15520, enhances Cpf1 with MLN4924 and inhibits ATM via Trichostatin A (Riesenberg & Maricic, 2018). Further, RS‐1 was shown to work additively with Scr7, a combination that led to a 74% increase in HR efficiency (Zhang et al., 2018).

## ASSAYS THAT QUANTIFY FREQUENCIES OF HR AND NHEJ THAT COULD BE EMPLOYED FOR SCREENING AND THE IDENTIFICATION OF YEAST‐SPECIFIC CHEMICALS

Several assays have been developed to measure the frequency of NHEJ or HR events in mammalian or yeast cells that have been used to either screen for molecules that bias the repair (Arras & Fraser, 2016; Chen et al., 2019; Ploessl et al., 2022) or to better understand the molecular mechanisms behind it (Bindra et al., 2013; Hussain et al., 2021). We highlight a few that are medium‐to‐high‐throughput compatible and that could be repurposed to measure or screen the effect of chemical agents on repair mechanisms in yeast. Other versions of these assays which are not specifically discussed here can be found in Pierce et al. (1999), Pastwa et al. (2009), Gunn and Stark (2012), Soong et al. (2015) and Wienert et al. (2020).

### Screening for DSB repair inhibition by measuring synergy between chemical inhibitors and DNA damaging agents—No genetics required

Small molecules that effectively inhibit DSB repair are toxic to cells at high concentrations (Arras & Fraser, 2016). As such, the minimal inhibitory concentration (MIC) of a given chemical should first be determined such that working concentrations with no growth effect can be chosen. Further, inhibition of NHEJ sensitises cells towards DNA‐damaging agents, as those are detrimental in case DSBs cannot be repaired. As such, Arras and Fraser (2016) used a simple assay measuring synergistic growth inhibition by a given small molecule in combination with a DSB‐inducing agent as a first indication that a small molecule affects DSB repair. The authors specifically test phleomycin, mercaptopurine and hydroxyurea as DNA‐damaging agents in combination with various potential DSB repair inhibitors in the yeast *C. neoformans*. This assay is a simple growth assay that can be performed on microtiter plates and does not require access to the genetics of the host.

### Integration/deletion assays based on selectable phenotypes—No prior access to genetics required

**FIGURE 3:**
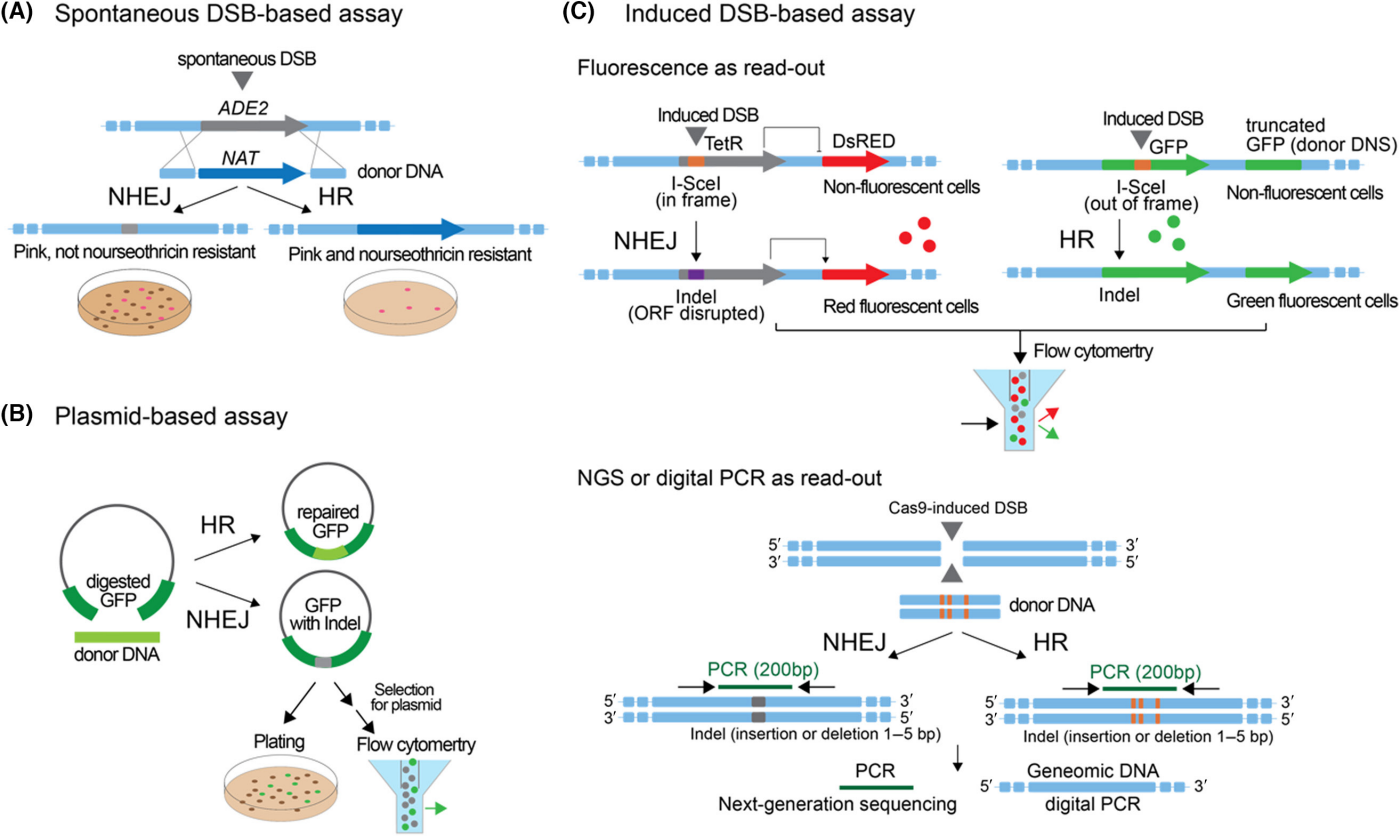
Potential medium‐ and high‐throughput (MTP/HTP) compatible assays to measure NHEJ/HR frequencies during small molecule screens in yeast. (A) *ADE2* encodes for phosphoribosylaminoimidazole carboxylase (Ade2) which catalyses a step in purine nucleotide biosynthesis. If deleted, a pink pigment accumulates in cells on media with limiting amounts of supplemented adenine (amounts that need to be titrated to receive a balance between cell growth and pigment production) that is visible to the naked eye and has been widely used as a read‐out. After a spontaneous DSB, HR with a donor DNA that encodes for a Nourseothricin resistance marker (*NAT*) yields pink colonies that are also resistant to Nourseothricin. Repair by NHEJ yields just pink colonies, as the Ade2 ORF can be disrupted due to Indels. This screen does not require a priori engineered yeast strains. (B) The plasmid‐based assay is based on the repair of a restriction‐digested GFP that is encoded on a plasmid with a donor DNA fragment during the co‐transformation of a given yeast with both fragments. The donor DNA can be generated by PCR using primer‐encoded homology arms of varying lengths. (C) A more quantitative measurement of NHEJ vs. HDR after an induced double‐strand break can be achieved via fluorescent devices and flow cytometry (upper panel) or NGS/digital PCR (lower panel). Note that in these systems, the cells need to express a genome‐editing system such as the I‐SceI endonuclease or a CRISPR/Cas9 system from a plasmid or the genome. A simpler version can be imagined where the I‐SceI recognition site is directly inserted out‐of‐frame into the RFP ORF. Subsequently, imperfect NHEJ repair with indels can potentially shift the frame to produce the correct RFP protein. Upper panel: The NHEJ reporter is based on a TetR repressor with an in‐frame I‐SceI site that represses a red fluorescent protein. In case the I‐SecI site is cut, error‐prone repair by NHEJ should yield a dysfunctional TetR and thus de‐repression of the red fluorescent protein reporter, quantifiable by flow cytometry. The HR sensor is based on a mutated GFP that carries an out‐of‐frame I‐SceI site to be repaired by a downstream encoded truncated GFP that serves as an intragenomic donor DNA, giving rise to a functional GFP gene, and GFP fluorescence measurable by flow cytometry. Lower panel: After cells have undergone repair of the Cas9‐generated break, the locus is PCR amplified around the break site (~200 bp product) and sequenced using Illumina‐based NGS. Alternatively, extracted genomic DNA is used as an input for droplet digital PCR (ddPCR). Bioinformatic analysis allows quantification of each repair event: NHEJ is thereby classified as short deletions ranging from 1 to 5 bp at the break site, and HR is characterised as a specific replacement with the repair oligo and its 3 mutations. A more detailed schematic of the resulting locus sequence after repair can be found in Hussain et al. (2021), Figure 1.

*ADE2* is a widely used non‐selective marker gene, as its deletion or disruption of the encoding ORF leads to visibly pink cells when grown on media with low amounts of adenine. *ADE2* encodes for the enzyme phosphoribosylaminoimidazole carboxylase (Ade2) which catalyses a step in purine nucleotide biosynthesis; if dysfunctional, a visibly pink pigment accumulates in cells. In combination with an antibiotic resistance marker such as the gene coding for Nourseothricin N‐acetyl transferase (NAT^R^), it can be used as a screen to distinguish the repair of a DSB in the *ADE2* locus by NHEJ or HR (Figure 3B). For example, cells can be transformed with a linear PCR‐generated DNA encoding for the *NAT* gene flanked by *ADE2‐directed* homology arms. If the DSB is repaired by HR, *NAT* gets inserted into the *ADE2* locus and cells are both pink and resistant to nourseothricin, while NHEJ‐repaired cells are just pink, in case the open reading frame is disrupted by Indels (Arras & Fraser, 2016).

Moreno‐Beltrán et al., applied a similar approach, just using the *URA3* gene as a maker, instead of *ADE2*. HR‐repaired DSB yield cells are both auxotrophic for uracil and nourseothricin resistant, while NHEJ‐repaired cells are just auxotrophic for uracil in case the Ura3 open reading frame is disrupted (Moreno‐Beltran et al., 2021).

### Plasmid‐based assays to quantify HR and NHEJ

Ploessl et al. developed an assay able to detect if a cut plasmid gets repaired by NHEJ or HR (Ploessl et al., 2022). It is based on transforming a plasmid where the encoded GFP has been cut with two restriction enzymes, leaving a linear plasmid with a truncated GFP. A donor DNA with 30 bp homology that encodes the missing piece is co‐transformed. Repair via HR using the donor DNA leads to the repair of the GFP and, consequently, green fluorescent transformants, while repair via NHEJ leads to simple plasmid circularisation and non‐fluorescent transformants (Figure 3). The results can be quantified by fluorescent/non‐fluorescent colony counting after plating on plasmid‐selective media using a transilluminator or by quantifying fluorescent/non‐fluorescent single cells using flow cytometry of plasmid selection in liquid media for several generations (higher throughput readout and less laborious). The assay could be performed in the presence or absence of various chemicals under investigation. The authors developed plasmids for transforming four yeast (*S. cerevisiae*, *K. marxianus, S. stipitis*, *Y. lipolytica*). The systems could likely be extended to other yeast, given that transformation protocols, a selection marker and a strong promoter driving GFP expression are available. Of note, the authors use rather short homology arms (30 bp) in comparison to other studies, and the sensitivity of the assay could potentially be enhanced by longer homology arms.

### Genomic DSB‐based assays for NHEJ and HR quantification—Prior plasmid transformation and/or genome engineering required

We highlight examples that allow quantifying NHEJ and HR after an induced DSB on the genome; they either use fluorescent proteins and flow cytometry for quantification or next‐generation sequencing (NGS) and droplet digital PCR (ddPCR) for quantification, the latter is cheaper and less bioinformatically demanding then NGS (Figure 3C).

Bindra et al. developed a fluorescent‐based assay (called EJ‐RFP) to detect NHEJ and verify it across various human cell lines (Bindra et al., 2013). It relies on inducing a genomic DSB by the rare‐cutting endonuclease I‐SceI (should be replaceable by a Cas‐system) and a genomically integrated reporter construct: A TetR repressor with an in‐frame I‐SceI site that represses the expression of a red fluorescent protein. In case the I‐SecI site is cut, error‐prone repair by NHEJ should yield a dysfunctional TetR and thus de‐repression of the red fluorescent protein reporter, quantifiable by flow cytometry (Figure 3C, upper panel). The authors combine their NHEJ‐detecting EJ‐RFP assay with a previously developed HR‐detecting ‘direct repeat green fluorescent protein’ (DR‐GFP) assay to quantify the ratio of both events in cells. In the DR‐GFP assay, the I‐SceI site has been integrated into a GFP gene, thereby disrupting its open reading frame (ORF). A truncated GFP fragment with the correct ORF sequence has been placed downstream in the construct and serves as an intragenomic donor DNA. Repair of the cleaved I‐SceI site by HR using the downstream fragment gives rise to a functional GFP gene, and GFP fluorescence can then be measured by flow cytometry.

Hussain et al. (2021) developed a Cas9‐based system that quantifies repair outcomes of NHEJ, HR, and a third alternative ‘backup pathway’, alternative end‐joining (Alt‐EJ), which has not been further discussed here. For the assay, cells need to be equipped with a Cas9/gRNA encoding system, either on a plasmid or genomically integrated. Cells are then co‐transformed with a single‐stranded and a double‐stranded ~200 bp repair oligonucleotide with three mutations. After cells have undergone repair of the Cas9‐generated break, the locus is PCR amplified and sequenced using Illumina‐based NGS. Alternatively, extracted genomic DNA is used as an input for droplet digital PCR (ddPCR). Bioinformatic analysis allows quantification of each repair event (Figure 3C, lower panel). This assay is HTP‐compatible, as many chemicals could be tested in parallel; it can be used to test repair at any locus that can be targeted by Cas9 and does not require genomically integrated reporter cassettes. The authors developed the assay in human cell lines, but it should also be applicable to yeast.

## DISCUSSION

The use of small molecules has proven promising for achieving higher success rates in the homology‐directed engineering of human cells without the need to modify the genome of the host. Not only does human engineering suffer from the fact that NHEJ dominates over HR, but it also impairs the implementation of modern genome engineering tools in many unconventional yeasts. Given the conserved proteins in repair pathways in mammal cells and yeast cells, researchers started exploring these mammalian‐optimised compounds for yeast engineering. Even though it has not been widely used yet, a few successful stories have been reported (Table 1, Supplementary Table S2).

The compound that has shown the most consistent success across yeast species and across various studies is the cell‐cycle synchroniser hydroxyurea, which arrests cells in the S phase of the cell cycle where HR dominates over NHEJ. But also, the calmodulin inhibitors W7 and Chlorpromazine, the ligase IV inhibitor Src7 and the Mre11 inhibitor Mirin showed promising results in yeast. Many compounds that showed success in humans have not yet been explored in yeast (Table 1, Supplementary Table S2). In addition, the combination of several small molecules with different modes of action could be explored, as successfully shown for mammalian cells (Riesenberg & Maricic, 2018; Zhang et al., 2018).

Importantly, in the instances where small molecules have been used in yeast to raise HR efficiency, the authors have not yet combined them with CRISPR/Cas systems but rather used pure homologue recombination‐based techniques in combination with rather long homology arms and selection markers. As such, combination with state‐of‐the‐art genome editing tools, especially those recently developed for unconventional yeast (Ploessl et al., 2022) could be very promising to generate broad‐species protocols that enhance HR. Also, natural strain‐to‐strain variability in HR efficiency needs to be considered when starting to engineer a new yeast species (e.g. testable via the assay outlined in Figure 3A). An isolate screen in *M. pulcherrima* exemplifies that only a few tested isolates were amenable to HR‐mediated gene insertions and enhancement via chemical inhibitors (Moreno‐Beltran et al., 2021).

Currently, available small molecules have been optimised for human cells. They have been either derived by HTP screens using available small molecule libraries and various purpose‐directed read‐out assays or via molecular modelling/docking using available 3D structures of human protein targets or available homologues. Both strategies could be employed to derive yeast‐specific molecules. We summarised several NHEJ/HR detection assays that could be employed in small‐molecule screens, as well as structure prediction tools that could be used to gain access to the 3D structures of repair proteins of various yeast species for docking and chemical refinement studies.

One aspect is still important to discuss: It cannot be ignored that a few of the small molecules employed in humans and yeast have been shown to increase HR efficiency in all cell types or yeast species. In fact, some small molecules can have non‐identical and even opposite effects on precise genome‐editing efficiencies across cell types. The reported efficiencies of the various chemicals in human cells are difficult to compare given the diversity of used cell lines, loci, small‐molecule concentrations, and protocols. As such, for yeast engineering, it would be meaningful to use standardised measurements and assays to measure HR‐related genome engineering, to gain lab‐to‐lab reusable protocols and to better understand the variability in success rates associated with a certain chemical agent.

Further, the potential toxicity of a given small molecule needs to be considered. For example, Song et al., found severe cell death in human‐induced pluripotent stem cells when treated with compound Scr7 (Song et al., 2016), while no adverse effects were observed in mouse embryos and several human cancer cell lines (Hu et al., 2018; Singh et al., 2015). Toxicity may be related to the concentration and to the specific cell type and as such the minimal inhibitory concentration should be determined before use in a new yeast, as exemplified for *C. neoformans* (Arras & Fraser, 2016). Besides that, some molecules show low solubility in DMSO as such compatible solvents need to be screened.

## AUTHOR CONTRIBUTIONS

**Sonja Billerbeck:** Conceptualization (equal); writing – original draft (equal); writing – review and editing (equal). **Min Lu:** Conceptualization (equal); writing – original draft (equal); writing – review and editing (equal).

## CONFLICT OF INTEREST STATEMENT

The authors declare no competing interests.

## Supporting information


Data S1.
