# Supplementary material for: Improving homology‐directed repair by small molecule agents for genetic engineering in unconventional yeast?—Learning from the engineering of mammalian systems
Source: Microb Biotechnol. 2024 Feb 20;17(2):e14398. doi: 10.1111/1751-7915.14398 (PMC10878012; doi:10.1111/1751-7915.14398)
Supplement: Supplementary file 1 — Data S1. [file MBT2-17-e14398-s001.docx]

**Supplementary Material**

**Improving homology-directed repair by small molecule agents for genetic engineering in unconventional yeast? - Learning from the engineering of mammalian systems.**

Min Lu^1^ and Sonja Billerbeck^1^*

^1^Molecular Microbiology, Groningen Biomolecular Sciences and Biotechnology Institute, University of Groningen, Groningen, The Netherlands

*Correspondence: [s.k.billerbeck@rug.nl](mailto:s.k.billerbeck@rug.nl)

**Supplementary Table 1. The concentration and current price of small molecules used in mammalian cells and yeast.**

| **Small molecules** | **Concentration in mammalian cells** | **Concentration in yeast** | **Price/mg^a^** |
| --- | --- | --- | --- |
| STL127705 | 2.5-100 µM | - | 22 € |
| STL127685 | 5 µM in hiPSCs | - | - |
| Compound 68 | 20 µM in MEF cells | - | - |
| W7 | 10-80 µM in MM cell lines | 13-130 µM,  20-80 µM | 2 € |
| Chlorpromazine | 5-20 µM in MM cell lines | 31-501 µM | 1.4 € |
| Scr7 | 0.01 ∼1 μM in A549 cell line | 150-1715 µM | 11 € |
| Scr130 | 7-21 µM in Reh cells | - | 11 € |
| Mirin | 50-400 µM in 1BR3hTERT | 91-1453 µM | 6 € |
| VE-822 | 0.5-2 μM in hiPSCs | - | 2 € |
| AZD7762 | 0.5-2 μM in hiPSCs | - | 5.6 € |
| Trichostatin A | 0.01 µM in hiPSCs | - | 24 € |
| MLN4924 | 0.5 μM in hiPSCs | - | 8 € |
| RS-1 | 7.5-15 μM in rabbit embryos | - | 4 € |
| Farrerol | 0.1-10 µM in HEK 293FT cells | - | 7 € |
| NSC15520 | 5 μM in hiPSCs | - | 14 € |
| Hydroxyurea | 2 mM in ES cell | 25-200 mM | 0.065 € |
| XL413 | 1-1000 μM K562-BFP cells | - | 18 € |
| PP1 | - | - | 20.6 € |
| Nocodazole | 200 ng/ml in ES cell | - | 3.5 € |

a: source: Selleckchem or MedChemExpress

| 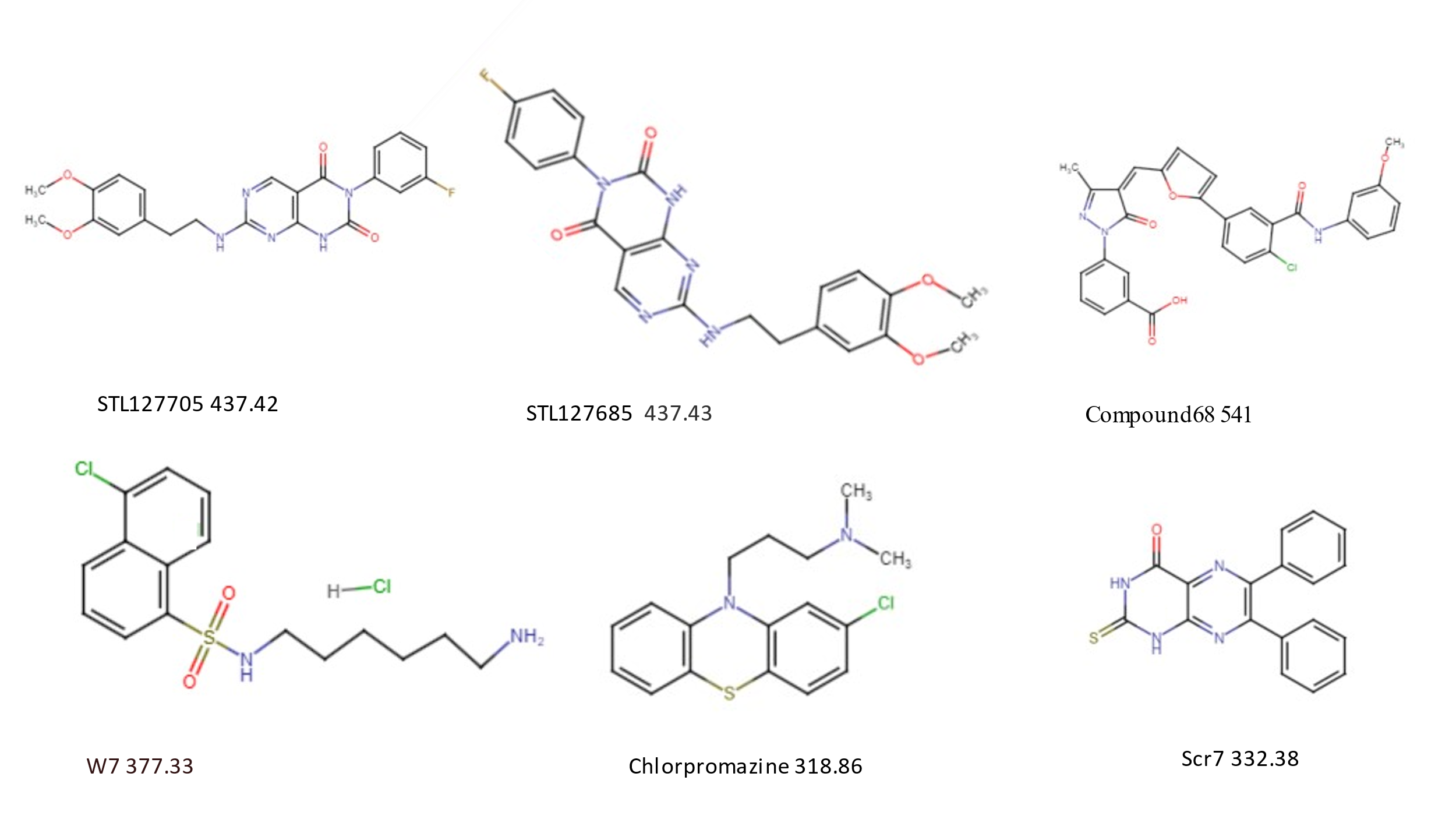 |
| --- |
| 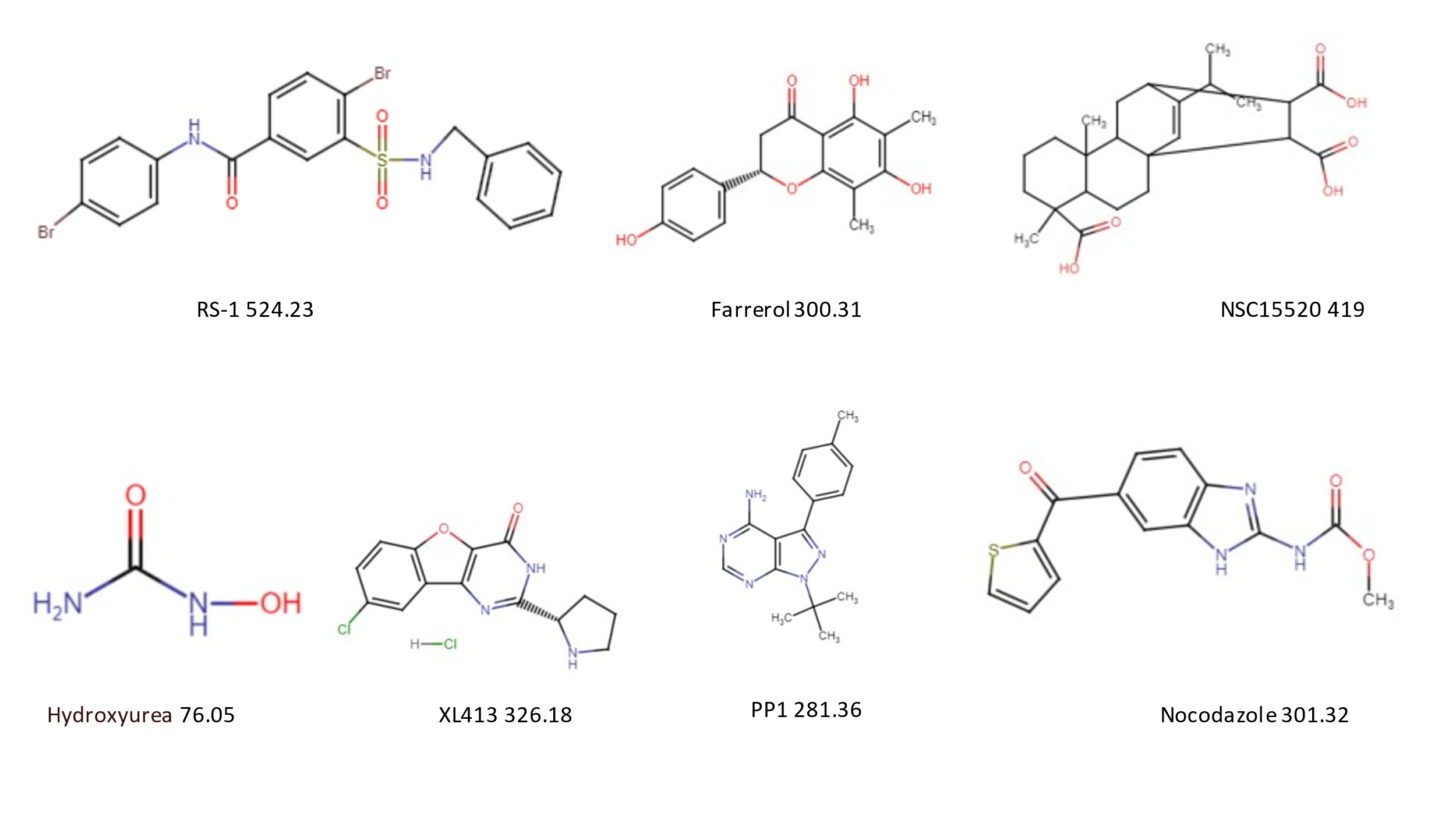 |
| 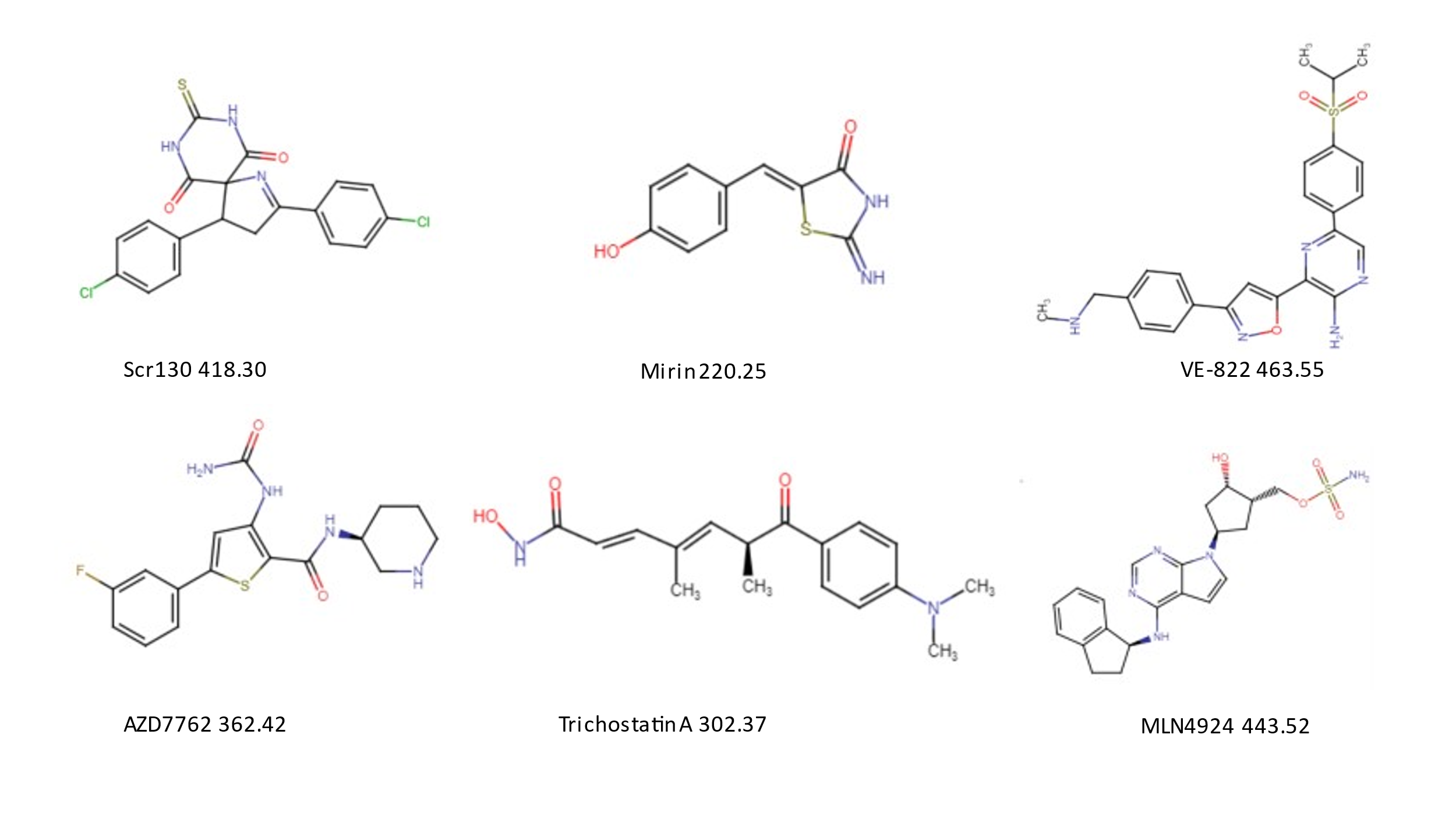 |

**Supplementary Figure 1:** Chemical structures and molecular weight (g/mol) of small molecules.

**Supplementary Table 2: Summary of small molecules discussed in the review and wether they have been used in yeast.**

| **Small molecules** | **Applied in yeast** |
| --- | --- |
| STL127705 | no |
| STL127685 | no |
| Compound 68 | no |
| Scr130 | no |
| VE-822 | no |
| AZD7762 | no |
| Trichostatin A | no |
| MLN4924 | no |
| RS-1 | no |
| Farrerol | no |
| NSC15520 | no |
| XL413 | no |
| PP1 | no |
| Nocodazole | no |
| aphidicolin | no |
| W7 | yes |
| Chlorpromazine | yes |
| Scr7 | yes |
| Mirin | yes |
| Hydroxyurea | yes |
